# Pro-Inflammatory Response of Bovine Lung Explant Induced by *Mycoplasma mycoides* subsp. *mycoides*

**DOI:** 10.3390/pathogens15030269

**Published:** 2026-03-03

**Authors:** Leruo Keokilwe, Giovanni Di Teodoro, Marta Di Federico, Massimo Ancora, Ivanka Krasteva, Gianluca Orsini, Cesare Camma, Fabrizia Perletta, Chiara Di Pancrazio, Mirella Luciani, Chandapiwa Marobela-Raborokgwe, Massimo Scacchia, Flavio Sacchini

**Affiliations:** 1 Department of Veterinary Services, Ministry of Lands and Agriculture, Gaborone Private Bag 0032, Botswana; 2 WOAH Reference Laboratory for Contagious Bovine Pleuropneumonia, Istituto Zooprofilattico Sperimentale dell’Abruzzo e del Molise “G. Caporale”, 64100 Teramo, Italy; 3 Department of Bioscience and Technology for Food, Agriculture and Environment, University of Teramo, 64100 Teramo, Italy; 4 Independent Researcher, Gaborone P.O. Box 202074, Botswana

**Keywords:** contagious bovine pleuropneumonia, *Mycoplasma mycoides* subsp. *mycoides*, bovine lung explant, pathogenesis, inflammation, cytokines, inflammatory mediators

## Abstract

Contagious bovine pleuropneumonia (CBPP) is a significant respiratory disease in cattle caused by *Mycoplasma mycoides* subsp. *mycoides* (*Mmm*). A better understanding of the pathogenesis of CBPP and the immune response of the host to infection will assist in the development of novel interventions to prevent disease progression. In this study, bovine lung explants (BLEs) were exposed to *Mmm* to investigate the upregulation and release of early inflammatory cytokines, mediators and receptors following tissue infection. Immunomodulatory molecules indicative of cell activation were investigated by immunoblotting on the BLEs and the tissue culture supernatants, and quantitative real-time PCR (RTq-PCR) was performed on the BLEs to determine the fold change in the expression of the respective mRNA. Immunoblotting indicated the production of inflammatory cytokines, mediators and receptors in *Mmm*-infected BLEs; however, this contrasted strongly with the mRNA expression profile, which did not show any significant fold increase. Infection of the BLEs with *Mmm* stimulated the production of some pro-inflammatory cytokines and mediators, including IL-1β, COX-2, 5-LOX and iNOS. Toll-like receptor proteins TLR2 and TLR4 were detected solely in the tissue culture supernatant of *Mmm*-infected BLEs. These receptors are considered to be involved in the recognition of *Mmm* by BLE tissue cells, thus triggering intracellular pathways that produce specific inflammatory cytokines and mediators, initiating the inflammatory response.

## 1. Introduction

Contagious bovine pleuropneumonia (CBPP) is an infectious respiratory disease in cattle caused by *Mycoplasma mycoides mycoides* (*Mmm*), a bacterium in the class *Mollicutes*, which has no cell wall and represents the smallest self-replicating unit [1]. CBPP has been eradicated in many parts of the world but persists in sub-Saharan Africa, with a negative economic impact on affected countries [2,3,4].

Clinical signs of acute disease include fever, rapid or difficult breathing, anorexia and cough exacerbated by exercise. These symptoms are ascribed to lung lesions characterised by fibrinous pleuropneumonia, often accompanied by a fibrinous pleural effusion. These classical CBPP pathological lesions represent acute lung inflammation, but knowledge of cells and immunomodulatory factors involved during *Mmm* infection is limited.

Investigation of CBPP pathogenesis has typically relied on a suboptimal in vivo challenge model using transtracheal intubation, sometimes combined with in-contact exposure of relatively large numbers of cattle [5]. This method raises ethical considerations, is costly and is not suited to elucidate the early events during disease pathogenesis, which shape clinical outcome [6]. In the absence of an animal model for CBPP, the use of ex vivo bovine respiratory organ cultures, in the form of bovine respiratory explants [6] and precision-cut lung slices [7], represents a viable alternative. These models are well suited to reveal the onset of inflammatory response and to gain an understanding of the cells involved and the inflammatory factors induced in the early stages of *Mmm* infection [6,7].

Pathogenic microorganisms, such as *Mmm*, or their components possess characteristic molecular patterns that constitutively differ from the host, known as pathogen-associated molecular patterns (PAMPs) [8,9]. Membrane-located toll-like receptors (TLRs) are the most important pattern recognition receptor (PRR) involved in the recognition of particular PAMPs [10,11]. Activation of TLRs launches the signal transduction pathways that ultimately cause nuclear factor (NF)-κB to activate expression of pro-inflammatory genes for cytokines and chemokines such as tumour necrosis factor-α (TNF-α), interleukin (IL)-1β, IL-6 and IL-8 [12,13,14]. They are mainly produced by monocytes and macrophages, although a broad spectrum of cell types such as epithelial cells and fibroblasts can produce them [15,16].

The eicosanoids are a group of bioactive signalling molecules that include leukotrienes and prostaglandins [17,18]. The 5-lipoxygenase (5-LOX) and cyclooxygenase (COX) pathways metabolise arachidonic acid into the leukotrienes and the prostaglandins, respectively [17], following the activation of cells by mechanical trauma or specific cytokines, growth factors and other stimuli [19].

Nitric oxide (NO) is derived from L-arginine in a metabolic pathway catalysed by a family of nitric oxide synthase (NOS) enzymes [20,21]. The NOS enzyme essentially involved in the production of NO in the inflammatory process is inducible NOS (iNOS) [20]. The expression of iNOS is not found in most resting cells but is induced in various inflammatory and tissue cells when exposed to microbial products such as lipopolysaccharide (LPS), lipoteichoic acid, peptidoglycan, bacterial DNA and intact bacteria or pro-inflammatory cytokines such as TNF-α, interferon (IFN)-γ and IL-1β [21,22,23].

The ex vivo explant model described by Di Teodoro et al. 2018 [6] was replicated to evaluate the early inflammatory response of bovine lung explants (BLEs) following *Mmm* exposure. Selected cytokines, inflammatory mediators and receptors, including TNF-α, IL-1β, IL-8, 5-LOX, COX-2, iNOS, TLR4 and TLR2, were targeted. This study aimed to use BLEs to characterise the inflammatory mediators produced by lung tissue infected with *Mmm*, with the goal of elucidating the contribution of lung tissue constituent and resident cells to CBPP pathogenesis.

## 2. Materials and Methods

### 2.1. Lung Sampling in the Abattoir

The right cranial lung lobe (n = 5) was aseptically sectioned from regularly slaughtered 18- to 24-month-old cattle within 20 min, and the tissue was immediately immersed in transport medium (TM, Table A1). The cattle belonged to herds located in the Abruzzo Region of Central Italy. The animals were clinically healthy, with no apparent lung lesions detected at post-mortem inspection. The samples were maintained in chilled conditions and transported to the laboratory within 2 h for further processing. Fresh lung samples from the abattoir (not stored in transport medium) were subjected to standard bacterial culture on blood and MacConkey agar under aerobic and CO_2_-enriched conditions to rule out any bacterial infection or contamination. These samples were further cultured on Mycoplasma agar and broth at 37 °C under CO_2_-enriched conditions to confirm the absence of *Mycoplasma* spp.

### 2.2. Bovine Lung Explant Preparation and Exposure to Mmm

A 1% low-gelling-temperature agarose (type VII-A agarose, Sigma-Aldrich, Saint Louis, MO, USA) at 37 °C was embedded in the lung parenchyma through the accessory bronchus, as previously described by Di Teodoro et al. 2018 [6]. Briefly, the lung lobe was then cooled to 4 °C for 20 min and then cut into 1 mm thick slices. Tissue samples of about 25 mm^2^ were transferred into 6-well plates, cultured in an air–liquid interface system and gently immersed in 1 mL of tissue culture medium (TCM, Table A2; Supplementary Materials, Figure S1).

**Figure A1:**
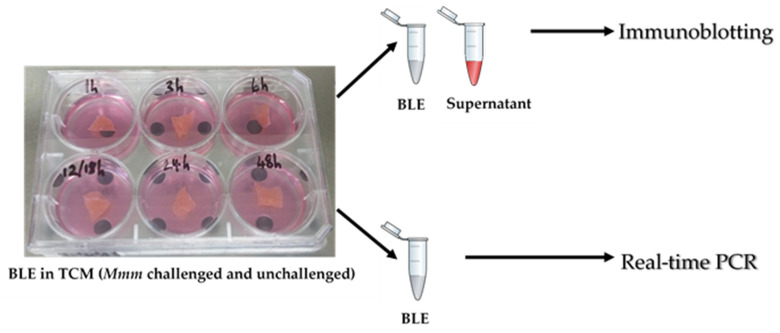
Diagram showing samples collected for immunoblotting and real-time PCR.

The BLEs were exposed to 1 mL of the *Mmm* “Caprivi” strain (1 × 10^9^ CFU/mL), a highly virulent strain from Namibia, prepared following a previously published protocol by Di Federico et al. 2020 [24]. Negative controls were prepared by adding 1 mL of TCM to the BLEs. The samples were incubated at 37 °C in 5% CO_2_ atmosphere, and the BLEs were extracted from the TCM (infected and non-infected) at 1, 3, 6, 18, 24 and 48 h post-*Mmm* inoculation. As illustrated in (Figure A1), the BLE samples were collected separately at each time point for protein (plain tube) and mRNA expression analysis (in RNAlater, Invitrogen, Bleiswijk, The Netherlands). In addition to the BLE, the tissue culture supernatant was also collected at each time point for protein expression analysis only. All samples were stored at −80 °C prior to testing.

### 2.3. RT-qPCR of BLEs and Gene Expression Analysis

The stored BLEs immersed in RNAlater were completely thawed, and each sample was cut to a weight of 0.5 g and homogenised using the TissueLyser machine (Qiagen, Hilden, Germany). The Direct Zoll RNA Kit (Zymo Research, Irvine, CA, USA) protocol was followed to extract RNA from the homogenate, which included a DNA digestion step (DNase I). Total RNA was quantified using the Qubit RNA HS (High Sensitivity) Assay Kit (Thermo Fischer Scientific, Eugene, OR, USA).

RT-qPCR analysis was performed, in duplicate, using the SuperScript™ III Platinum™ One-Step RT-qPCR Kit (Invitrogen), according to the manufacturer’s instructions, to quantify the relative expressions of a panel of seven target genes involved in the inflammatory process. Primers and TaqMan probes (Eurofins Genomics, Murcia, Spain) targeting IL-1β, 5-LOX, iNOS, TLR4 [24], IL-8, TNF-α [25] and COX-2 [26] were used as previously described by Di Federico et al. 2020 [24] and shown in Table 1. Briefly, retro-transcription and amplification were carried out in 96-well plates, distributing 5 µL of RNA suspension in 20 µL of reaction volume using the QuantStudio 7 Flex Real-Time PCR System instrument (Applied Biosystem, Carlsbad, CA, USA) under the following thermal cycling conditions: 15 min. at 50 °C (retro-transcription), 2 min at 95 °C (Taq polymerase activation) and 40 cycles of 15 s at 95 °C and 30 s at 60 °C (amplification reaction).

Target gene expression was evaluated against the β-actin (β-ACT) [27] housekeeping gene using the 2^(−ΔΔCT)^ method [28].

Statistical analyses were performed using a one-sample *t*-test (unilateral) compared with a theoretical mean of 1. The test was considered significant when the observed mean was greater than 1 with a *p*-value < 0.05. Delta = 1 was set to indicate no difference in gene expression between the two experimental conditions considered.

### 2.4. Immunoblotting

The BLEs were cut to a weight of 50 µg, added to a solution of tissue lysis buffer (CelLytic^TM^ MT, Sigma-Aldrich) and protease inhibitor (cOmplete^TM^, EDTA-free, Roche, Mannheim, Germany) and then homogenised (Precellys^®^ 24, Bertin Corp, Montigny le Bretonneux, France). The tissue lysate was mixed in a 50:50 solution with buffer and then heat-treated at 70 °C for 10 min. The BLEs were then ready for the immunoblotting test, for which the tissue culture supernatant was used directly.

In total, 15 µL of the BLEs (as prepared above) and 8 µL of tissue culture supernatant for both *Mmm*-challenged and unchallenged conditions, in triplicate, were directly separated with electrophoresis using a NuPAGE 4–12% Bis-Tris gel (Novex, Life Technologies, Bleiswijk, The Netherlands) at a constant voltage of 200 V. Samples were then placed onto iBlot2 NC stacks with the gel layered on the nitrocellulose membrane (Life Technologies), and the protein transfer was effected with the iBlot2^®^ Dry Blotting System (Life Technologies) at 20 V for 1 min, 23 V for 4 min and 25 V for 2 min. Membranes were blocked with PBS containing 0.05% Tween 20 (PBST) and 5% skim milk for 2 h at room temperature. The skim milk was discarded, and the membranes were incubated overnight at room temperature with specific antibodies: Rabbit anti-bovine, including IL-1β (Bio-Rad, Hercules, CA, USA), 5-LOX (Novus Biologicals, Centennial, CO, USA), COX-2 (Merck Millipore, Darmstadt, Germany), TLR4 (Boster Biological Technology; Pleasanton, CA, USA) and iNOS (Enzo Life Sciences, Long Island, NY, USA) and Mouse anti-bovine, including IL8 polyclonal (anti-IL8 antibody ab193818, Abcam, Cambridge, UK) and monoclonal (anti-bovine IL8 (CXCL8) mAb MT8H6, Mabtech AB, Nacka Strand, Sweden) and TNFα (Bio-Rad). All antibodies were diluted to 1:1000 in PBST containing 2.5% skimmed milk.

After washing three times with PBST and once with 10% skimmed milk in PBST for 10 min at room temperature, the membranes were incubated for 1 h at room temperature with goat anti-rabbit IgG-HRP (Bio-Rad) diluted to 1:3000, or anti-mouse IgG-HRP (GE Healthcare) diluted to 1:8000 in PBST containing 2.5% skimmed milk. After five washes with PBST for 5 min and one final wash with PBS for 10 min, the membranes were developed for chemiluminescence detection with the ECL Select^TM^ Western blotting detection kit (Amersham^TM^, GE Healthcare, Little Chalmont, UK). Images were acquired using ChemiDoc MP (Bio-Rad) and Image Lab Software, version 4.0.1 (Bio-Rad).

## 3. Results

### 3.1. RT-qPCR

**Figure 1:**
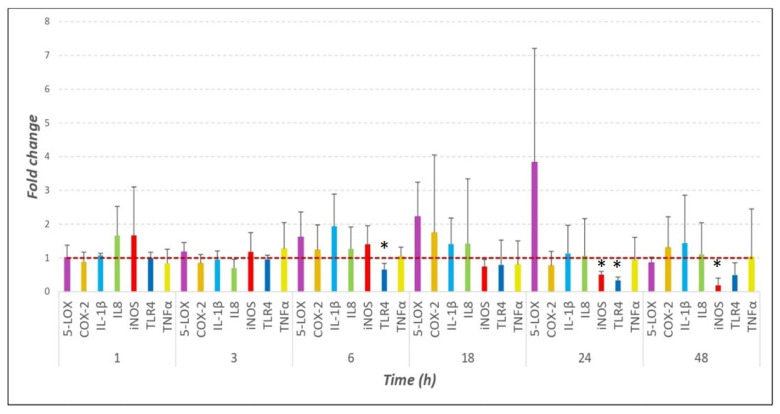
Fold change in mRNA levels quantified using RT-qPCR (normalised to β-ACT, calibrated to the challenged condition) after BLE exposure to *Mmm* at all considered time points. * *p* < 0.05.

Generally, the RT-qPCR results indicate that the gene expression levels of most pro-inflammatory factors considered in this study were not significantly modulated following BLE exposure to *Mmm* (Figure 1).

COX-2, IL-8 and TNFα showed fold change values of around 1-fold (close to the delta value) for all time intervals considered, starting from 1 h post-challenge. Similarly, IL-1β showed a homogeneous gene expression profile very close to the delta value (1-fold), except for the 6 h time point, where the fold change reached its maximum value (1.9-fold). However, the differences between the two compared conditions (challenged and unchallenged BLEs) recorded at any of the time points tested for these inflammatory factors were not statistically significant (*t*-test, *p* > 0.05).

The 5-LOX mRNA expression levels in challenged BLEs were considerably higher than the control values (unchallenged BLEs) at 18 h (2.2-fold) and 24 h (3.8-fold); however, these differences were not statistically significant (*t*-test, *p* > 0.05).

TLR4 and iNOS showed a different expression profile characterised by decreasing fold change values. In fact, TLR4 recorded fold change values consistently lower than delta value (1-fold), and its mRNA expression level decreased over time starting from 1 h post-challenge. Moreover, at 6 h (0.65-fold; *p* = 0.033) and 24 h (0.34-fold; *p* = 0.001), the differences recorded between the challenged and unchallenged BLEs were statistically significant (*t*-test, *p* < 0.05). The iNOS mRNA expression levels also decreased over time, reaching their lowest value at 48 h post-treatment (0.19-fold). Statistically significant differences (*t*-test, *p* < 0.05) in the iNOS mRNA expression levels between the two considered conditions (challenged and unchallenged BLEs) were observed at the 24 h (*p* = 0.012) and 48 h (*p* = 0.005) time points.

### 3.2. Immunoblotting of BLEs

**Figure 2:**
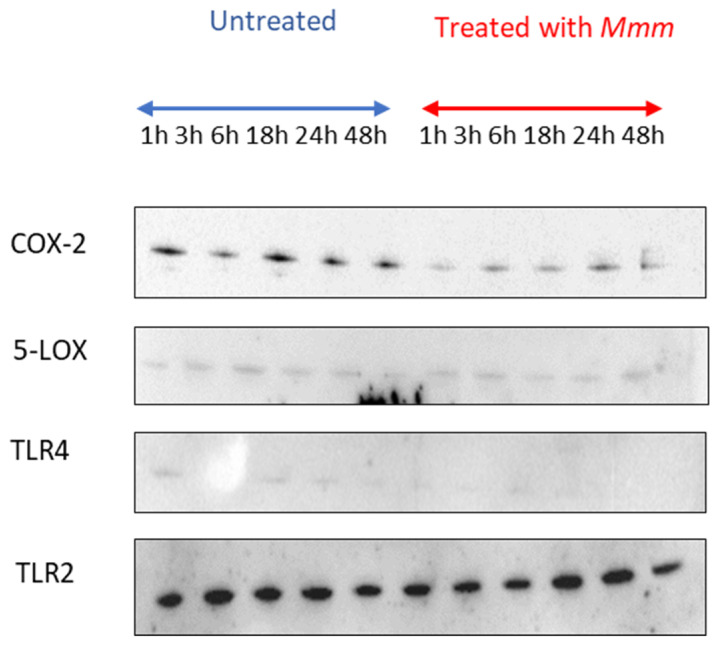
Immunoblotting results for *Mmm*-challenged and unchallenged BLEs.

Bands were detected for COX-2, 5-LOX and TLR2 at all time points for both the unchallenged and challenged BLEs (signifying constitutive production). Bands were detected for TLR4 (very faint) at all time points for the unchallenged BLEs and from 1 h to 18 h for the challenged BLEs (Figure 2). No bands were detected for TNF-α, IL-1β, IL-8 or iNOS.

### 3.3. Immunoblotting of Supernatant

**Figure 3:**
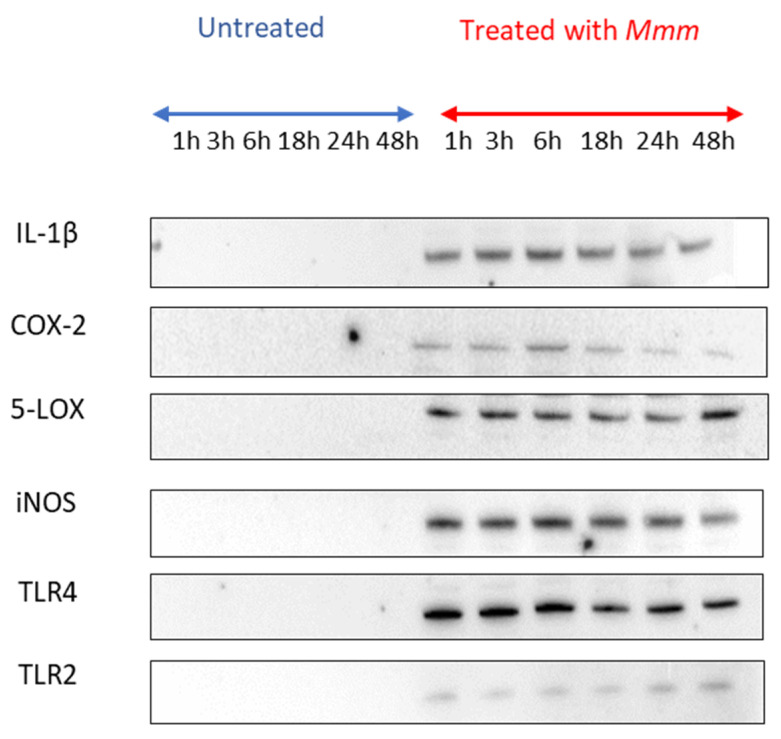
Immunoblotting results for tissue culture supernatant from *Mmm*-challenged and unchallenged BLEs.

Bands were detected only from the *Mmm*-challenged supernatant at all time points for IL-1β, COX-2, 5-LOX, iNOS, TLR4 and TLR2, suggesting their production and accumulation in the supernatant (Figure 3). No bands were detected for TNF-α or IL-8.

### 3.4. Limitations of Immunoblotting

CBPP-infected lungs from previous field samples were used as positive controls. The interpretation of immunoblotting data, particularly iNOS and TLR2, is constrained by the lack of optimal positive controls. In fact, for IL-8 no positive results were recorded from the controls.

While consistent patterns across replicates and time points support our conclusions, future studies should employ recombinant proteins or knockdown/knockout controls to definitively confirm antibody specificity.

Furthermore, we cannot fully rule out the potential contribution of *Mmm*-derived antigens to the band detected in the supernatant, although the use of antibodies raised against bovine targets makes this less likely.

## 4. Discussion

Inflammatory cytokines are rapidly induced and expressed early in the process of infection, such as with mycoplasmas [15,16,29], and the primary cytokines produced include TNF-α, IL-1β and IL-6 [16]. Contrary to what may have been expected, there was no detection of TNF-α production in the *Mmm*-infected BLEs. Macrophages are the predominant cell type to produce TNF-α [8,30], and this production has been observed in macrophages stimulated with *Mmm* [31]. Elevated levels of TNF-α were also detected in the lung tissue [32] and plasma [33] of naturally *Mmm*-infected animals.

Alveolar, intravascular and interstitial macrophages are heterogeneous in function [34]. Resident alveolar macrophages form a self-replenishing subset augmented at a basal level by blood-derived monocytes. In mice, these resident cells are subdued with low production of inflammatory cytokines. However, an inflammatory proponent can induce a marked influx of monocytes, referred to as exudative macrophages in the lung tissue, which then produce TNF-α [34]. One drawback of the BLE model is that the circulation is cut off and the infiltration of immune cells attracted to the inflammatory site is absent [7,35]. The lack of TNF-α detection in the BLEs may indicate that exudative macrophages account for the majority of TNF-α production, while other components are not significant producers of TNF-α at the onset of *Mmm* infection.

In the BLEs stimulated by *Mmm*, IL-1β was produced and predominantly secreted. IL-1β was produced by embryonic bovine lung cells exposed to *Mmm*-derived lipid-associated membrane proteins and detected in the supernatant [36]. These results are comparable to those of the current study, where IL-1β was produced and released from BLEs after exposure to *Mmm*, supporting the role of IL-1β as one of the primary cytokines in the early inflammatory response to *Mmm* in the bovine lung.

IL-8 is a chemokine that is responsible for the chemotaxis and activation of neutrophils and is involved in CBPP pathogenic mechanisms [24,37,38]. The detection of IL-8 in the BLE cell lysate or in culture supernatant could not be substantiated in this study. The CBPP-infected lung that was used as a positive control for IL-8 yielded a negative immunoblot result; therefore, no conclusion can be drawn about *Mmm*’s effects on the production of this chemokine.

The IL-8 mRNA expression profile yielded no significant fold increase. This differs from recent studies, where there was a significant increase in IL-8 mRNA expression in PMNs exposed to *Mmm* [24]. One possible explanation is that the stimulus caused by *Mmm* in the infection model set up was not sufficient to activate the pathway for IL-8 production. BLEs contain few alveolar macrophages and very low or no PMNs, which are major IL-8 producers. Therefore, even though epithelial cells, airway smooth muscle cells and endothelial cells can also release IL-8, exposure of these cells to *Mmm* did not cause a detectable increase in IL-8 mRNA expression.

COX-2 and 5-LOX are constitutively expressed at low levels in normal lungs and induced at high levels during inflammation [39]. This was evident in the BLEs, as both COX-2 and 5-LOX were detected in both infected and uninfected BLEs. The production of COX-2 and 5-LOX was detected in the supernatant, which could be attributed to exposure of the BLE to *Mmm*, as this was not evident from the uninfected BLEs. This indicates that *Mmm* infection induces the production of COX-2 and 5-LOX.

The enzyme iNOS is not normally expressed in resting cells, but it is induced by microbial products (e.g., LPS, dsRNA) or pro-inflammatory cytokines (e.g., TNF-α, IL-1, IFN-γ) [20,21]. No iNOS was detected in the non-exposed BLEs, which is in agreement with the typical lack of expression of iNOS in resting cells. However, the finding of iNOS only in the supernatant of *Mmm*-exposed BLEs strongly suggests that this enzyme was produced in response to infection directly or the production of cytokines, such as IL-1β.

TLR4 is present in the resting cell, where it shuttles between the Golgi apparatus and the cell membrane and is translocated to the cell surface in response to LPS exposure. The constitutive expression of TLR4 on the cell surface of human alveolar macrophages is low and is enhanced by exposure to LPS [40]. Similarly, in the non-exposed BLEs, the presence of TLR4 was low, as demonstrated by weak to no detection. Contrary to expectations, TLR4 was weakly detected or not detected at all in the *Mmm*-exposed BLEs. A distinct presence of TLR4 was only detected in the supernatant of the *Mmm*-exposed tissue cultures, not in that of non-exposed BLEs. Generally, TLRs are classified as transmembrane glycoproteins [12,41] and, in this case, are expected to be associated with the BLE tissue cells. Therefore, finding TLR4 in the supernatant suggests disintegration of the cellular membrane due to *Mmm*-induced cell death, such as the pyroptosis described below, leading to its passive release.

The mycoplasma ligands for TLR4 are unknown, as mycoplasmas lack a cell wall, which normally includes LPS, for which TLR4 is a receptor [42,43]. Lipoproteins are considered to be the main components of intact mycoplasmas that activate the inflammatory response during infection [44], and they are recognised by TLR2 complexed with TLR1 or TLR6 [42,45,46]. Similar to TLR4 in this study, TLR2 was constitutively expressed in some of the BLEs and was detected in the supernatant of the *Mmm*-exposed BLEs only. This indicates that TLR2 protein production occurred in the *Mmm*-infected BLE. *Mycoplasma ovipneumoniae* has also been shown to induce the production of TLR4 and the upregulation of TLR2 in sheep bronchial epithelial cell cultures [47].

For all these genes, IL-1β, COX-2, 5-LOX, iNOS and TLR4, no correlation was found between mRNA expression and protein detection. Complex and varying regulatory mechanisms post-transcription and/or post-translation can be responsible for the poor correlation observed [48]. The mRNA of many inflammatory mediators, cytokines and chemokines is unstable. This is attributed to the presence of *cis*-acting adenine and uridine-rich (AU-rich) elements, or AREs, in their 3′ untranslated (UTR) regions. *Trans*-acting factors, such as RNA-binding proteins, attach to these AREs and affect the stability and/or translation of the mRNA [49].

IL-1β is an unconventional cytokine in that it is expressed as a precursor, pro-IL-1β, which requires maturation for activity. Furthermore, unlike most cytokines, it does not possess a signal sequence to direct its exit from the cell via secretory vesicles [50]. The inflammasomes are cytosolic multiprotein complexes that are assembled in response to signals indicative of cell injury, stress or infection. These inflammasomes incorporate and regulate caspase-1, a cysteine protease that induces the cleavage of pro-IL1β to its active form and enables its secretion [50,51]. Pyroptosis is an inflammatory form of cell death associated with caspase-1 activity and leads to a type of osmotic cell lysis and release of pro-inflammatory cellular content [51,52]. This cell death process could account for the presence of the above inflammatory mediators, cytokine and receptors in the supernatant.

## 5. Conclusions

For the first time, the cytokines and pro-inflammatory mediators IL-1β, 5-LOX, COX-2 and iNOS produced by lung tissue in early *Mmm* infection are reported using the BLE model. TLR2 and TLR4 production was also observed, indicating the possible role of these receptors in the recognition of *Mmm* infection in the host or their possible role in modulating the immune response. This provides insight into the early pathogenesis of CBPP, confirming that *Mmm* elicits the production of pro-inflammatory cytokines and mediators in BLEs as initiating factors of the immune response. Although the individual roles of specific cells in BLEs (bronchiolar epithelial cells, alveolar epithelial cells (AECs) type I and II, resident alveolar macrophages and vascular endothelial cells) remain to be delineated, these data provide the first evidence that lung parenchymal cells (e.g., epithelial and endothelial cells) contribute to the onset of the inflammatory response, which is then amplified by recruited immune cells. Future investigations should aim to identify the contribution of each cellular population in BLEs, employing immunohistochemical and single-cell approaches to localise cytokine production and receptor expression. Moreover, the integration of transcriptomic and proteomic analyses could provide a more comprehensive overview of the host–pathogen interaction and reveal novel biomarkers of early CBPP infection.

## Figures and Tables

**Table 1:** Sequences of PCR primers and TaqMan probes.

Target Gene	Primer	Sequence (5′-3′)	Probe	Probe Sequence (5′-3′)	Accession Number	Reference
β-ACT	ACT-F	CAGCACAATGAAGATCAAGATCATC	ACT-1081-Probe	TCGCTGTCCACCTTCCAGCAGATGT	AY141970	[27]
	ACT-R	CGGACTCATCGTACTCCTGCTT				
COX-2	COX_2_Fw	CCAGAGCTGCTTTTCAACCAA	COX_2_Probe	TCCAGTACCAGAACCGT	AF031698	[26]
	COX_2_Rev	AGCGTGTTAAACTCAGCAGCAA				
5-LOX	5ALOX_Fw	GAGATGGGCAAGCGAAGTTG	5ALOX_P	ACCAAATTCACGTTCTCAAGCAGCACAGA	NM_001192792	[24]
	5ALOX_Rev	TTTTGCCGTGTCTCCAGTTCT				
IL-1β	IL1B_F	ACCTTCATTGCCCAGGTTTCT	IL1B_Probe	CAACCGTACCTGAACCCATCAACGAAA	EU276067	[24]
	IL1B_R	ACAGCTCATTCTCGTCACTGTAGTAAG				
iNOS	iNOS_Fw	TCTGCAGACACGTGCGTTATG	iNOS_P	ACAACGGCAACATCAGGTCGGCC	AJ699400	[24]
	iNOS_Rev	TCCAGACCCGGAAGTCATG				
TLR4	TLR4_F	TGCGTACAGGTTGTTCCTAACATT	TLR4_Probe	AAAATCCCCGACAACATCCCCATATCAA	KX138607	[24]
	TLR4_R	CTGGAGAAGTTATGGCTGCCTAA				
IL8	IL8.177F	CACTGTGAAAAATTCAGAAATCATTGTTA	IL8.214P	AATGGAAACGAGGTCTGCTTAAACCCCAAG	S74436	[25]
	IL8.282R	CTTCACCAAATACCTGCACAACCTTC				
TNFα	TNF.338F	TCTTCTCAAGCCTCAAGTAACAAGT	TNF.367P	AGCCCACGTTGTAGCCGACATCAACTCC	Z14137	[25]
	TNF.440R	CCATGAGGGCATTGGCATAC				

## Data Availability

The original data presented in the study are openly available in [FAIRDOMHub] at [DOI: 10.15490/fairdomhub.1.datafile.8086.1/https://fairdomhub.org/data_files/8086?version=1 (created on: 30 October 2025)] and [DOI: 10.15490/fairdomhub.1.datafile.8178.1/https://fairdomhub.org/data_files/8178?version=1 (created on: 19 November 2025)] [53].

## Supplementary Materials

The following supporting information can be downloaded at https://www.mdpi.com/article/10.3390/pathogens15030269/s1, Figure S1: Illustration of bovine lung explant (BLE) in tissue culture medium (TCM).

## Abbreviations

The following abbreviations are used in this manuscript:

CBPP	Contagious bovine pleuropneumonia
*Mmm*	*Mycoplasma mycoides* subsp. *mycoides*
BLE	Bovine lung explant
RT-qPCR	Quantitative reverse transcription polymerase chain reaction
TNF-α	Tumour necrosis factor-alpha
IL-1β	Interleukin-1 beta
IL-8	Interleukin-8
COX-2	Cyclooxygenase-2
5-LOX	5-Lipoxygenase
iNOS	Inducible nitric oxide synthase
TLR2	Toll-like receptor 2
TLR4	Toll-like receptor 4
β-ACT	Beta-actin
TM	Transport medium
TCM	Tissue culture medium
CFU	Colony forming unit
PMNs	Polymorphonucleocytes
LPS	Lipopolysaccharide

## Appendix A

### Appendix A.1

**Table A1:** Composition of transport medium for lung tissue.

Transport Medium (TM)	
Material	Quantity
Sterile PBS (pH 7.2)	1000 mL
Ampicillin (Sigma-Aldrich, Saint Louis, MO, USA)	1 g
Amphotericin B (Sigma-Aldrich)	2 mg

### Appendix A.2

**Table A2:** Composition of culture medium for BLEs.

Tissue Culture Medium (TCM)	
Material	Quantity
Dulbecco’s modified Eagle medium (DMEM) (Gibco, Grand Island, NY, USA)	1000 mL
Glutamax (Gibco)	300 mg
Bovine insulin (Sigma-Aldrich, Saint Louis, MO, USA)	1.5 mg
Hydrocortisone (Sigma-Aldrich)	0.3 mg
Vitamin A (Sigma-Aldrich)	0.5 mg
Ampicillin (Sigma-Aldrich)	300 mg
Amphotericin B (Sigma-Aldrich)	2 mg
