# Supplementary figures and images for: Pro-Inflammatory Response of Bovine Lung Explant Induced by Mycoplasma mycoides subsp. mycoides
Source: Pathogens. 2026 Mar 3;15(3):269. doi: 10.3390/pathogens15030269 (PMC13028708; doi:10.3390/pathogens15030269)

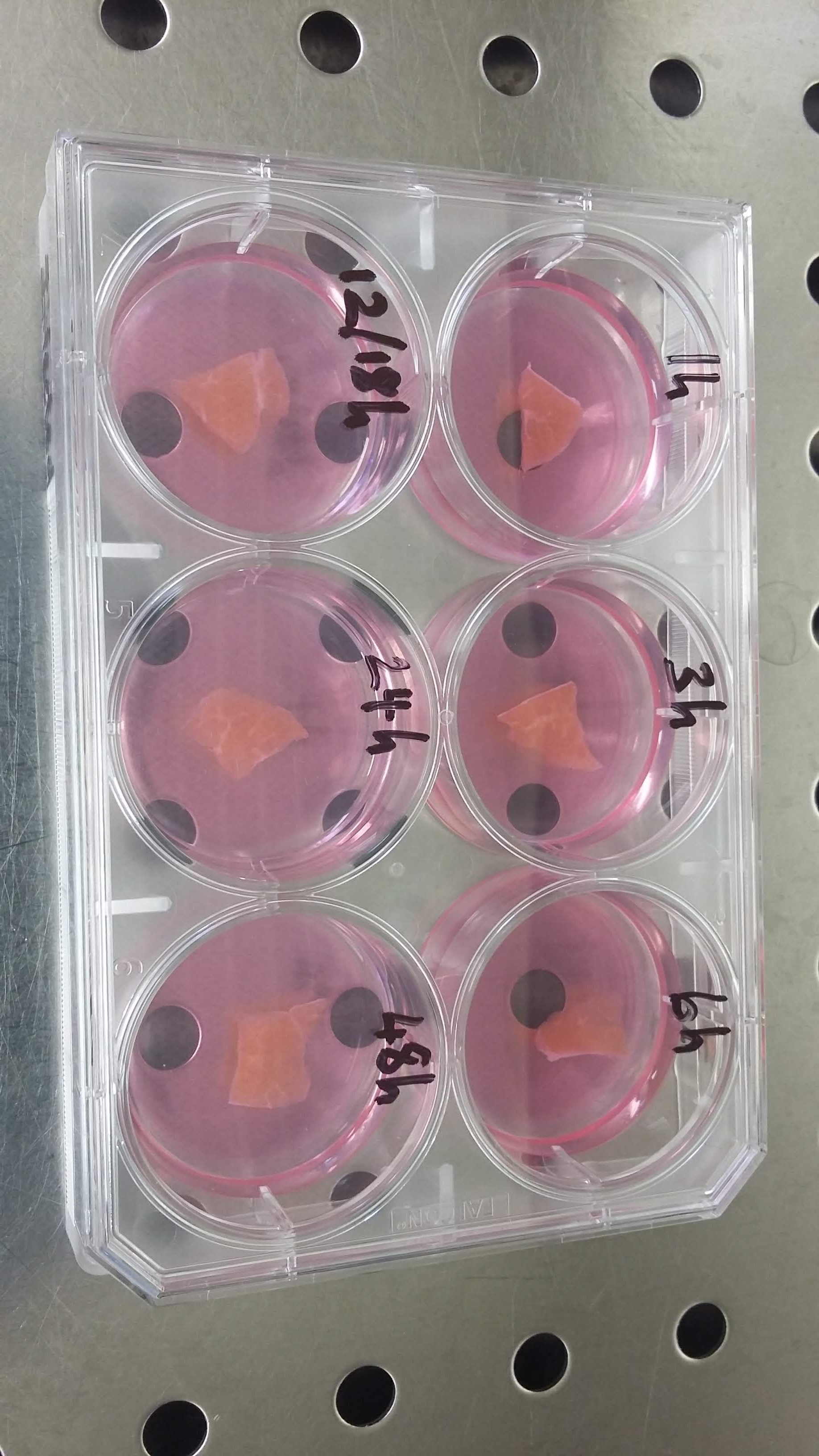

Supplement: Supplementary file 1 [file pathogens-15-00269-s001.zip › pathogens-4033057-supplementary.jpg]
